# Mental health help-seeking among individuals with breast cancer: A qualitative exploration of women’s and healthcare practitioners’ perspectives

**DOI:** 10.1371/journal.pone.0342002

**Published:** 2026-01-30

**Authors:** Nurdiana Mohammad Hussin, Nik Ruzyanei Nik Jaafar, Idayu Badilla Idris, Azmawati Mohammed Nawi

**Affiliations:** 1 Department of Public Health Medicine, Faculty of Medicine, Universiti Kebangsaan Malaysia, Kuala Lumpur, Malaysia; 2 Department of Psychiatry, Faculty of Medicine, Universiti Kebangsaan Malaysia, Kuala Lumpur, Malaysia; BRAC Business School, BRAC University, BANGLADESH

## Abstract

Individuals with breast cancer (BC) experience significant psychological distress, yet their utilization of mental health services remains low. This study identified key factors influencing help-seeking behavior through integrated Theory of Planned Behavior (TPB) and Health Belief Model (HBM) frameworks. We conducted in-depth interviews (IDIs) with eight BC patients and nominal group technique (NGT) with six health professionals, followed by Fuzzy Delphi Method (FDM) to assess expert consensus. The IDIs revealed that the individuals with BC recognized the value of professional psychological support but were reluctant to engage with these services personally. The participants identified emotional thresholds for help-seeking, expressed preference for informal support networks, and demonstrated varied understanding of mental health professional roles. The FDM evaluation demonstrated strong expert consensus across all assessed elements, particularly those related to emotional support mechanisms. Three barrier categories emerged: individual factors (mental health literacy, autonomy preferences), social factors (family support, cultural stigma), and systemic factors (healthcare integration gaps). Expert consensus exceeded 80% agreement across all domains.This study identified a complex interplay between individual psychological barriers and systemic factors affecting mental health help-seeking among individuals with BC. Effective interventions must address psychological barriers and healthcare delivery factors while respecting individual autonomy in coping choices. A multi-level approach targeting individual education, family support systems, and healthcare integration is recommended to improve mental health service utilization among individuals with BC.

## Introduction

The psychological impact of breast cancer (BC) extends beyond physical manifestations, with 30–40% of patients experiencing clinically significant anxiety and depression [1,2]. Despite this substantial psychological burden, mental health service utilization among this population remains consistently low, where approximately half of those experiencing distress do not seek professional assistance [3,4]. This treatment gap represents a significant public health concern, as untreated psychological distress is associated with reduced treatment compliance, diminished quality of life, and potentially increased mortality rates among individuals with BC [5].

Psychological distress manifests across the cancer trajectory, with patients facing fear of recurrence, body image changes, fertility concerns, financial burden, and social role disruption [2]. Distress peaks at diagnosis, during active treatment, and at treatment completion when support often diminishes [4]. Moreover, the long-term psychological sequelae of BC can persist for years after treatment completion, with survivors reporting elevated rates of anxiety, depression, and post-traumatic stress symptoms compared to the general population [6]. These findings underscore the critical importance of understanding and addressing barriers to mental health help-seeking in this vulnerable population.

Despite the well-established prevalence and consequences of psychological distress among individuals with BC, significant disparities exist in mental health service utilization. International studies have revealed that mental health support uptake varies considerably across different healthcare contexts, influenced by factors including healthcare system structure, cultural attitudes toward mental illness, availability of psycho-oncology services, and socioeconomic determinants of access [7,8]. In many settings, including Malaysia, mental health services for cancer patients remain fragmented, with limited integration between oncology and mental health care [9]. The absence of routine psychological screening in oncology settings, coupled with insufficient psycho-oncology specialist availability, creates structural barriers that compound individual-level obstacles to help-seeking [10]. Understanding the interplay between systemic healthcare factors and individual psychological barriers is therefore essential for developing contextually appropriate interventions.

Previous research has primarily used quantitative methodologies to identify factors like stigma and mental health literacy (MHL) influencing help-seeking [11,12]. While valuable for establishing prevalence and identifying associations, these approaches cannot fully capture the complex interplay of personal beliefs, social influences, and contextual factors that shape help-seeking decisions in real-world settings [13]. The field is increasingly recognizing that quantitative methods alone are insufficient for capturing the complex realities of those facing mental health challenges [14]. Understanding these complex factors influencing mental health help-seeking behavior through in-depth qualitative exploration is essential for developing effective interventions to address the treatment gap.

The conceptualization of mental health help-seeking has evolved considerably over recent decades, with researchers recognizing it as a complex, multi-stage process rather than a single binary decision. [15] proposed a comprehensive conceptual framework identifying help-seeking as encompassing awareness of need, intention to seek help, actual help-seeking behavior, and willingness to disclose and receive help. This process-oriented understanding highlights that barriers can occur at any stage, from initial problem recognition through to sustained engagement with services.

[16] examining gender differences in psychological help-seeking attitudes in Turkiye found that sociodemographic factors, mental health status, and access to support networks significantly predict help-seeking behavior, with notable gender disparities in willingness to engage with mental health services, supporting the TPB’s emphasis on subjective norms and perceived behavioral control as critical determinants.

Recent systematic reviews have documented that mental health stigma operates at multiple levels—public stigma, self-stigma, and structural stigma—each uniquely impeding help-seeking pathways [17]. Moreover, the COVID-19 pandemic has necessitated reconceptualizing help-seeking to incorporate online and tele-mental health modalities, revealing both opportunities for increased accessibility and new barriers related to digital literacy and privacy concerns ([18,19]. Understanding these conceptual developments is essential for designing interventions that address the full spectrum of help-seeking barriers rather than focusing narrowly on single determinants.

Contemporary understanding recognizes that help-seeking barriers operate at multiple ecological levels: individual, interpersonal, community, and systemic. At the individual level, MHL plays a foundational role, though literacy alone is insufficient; self-stigma represents a distinct barrier where individuals internalize negative societal attitudes about mental illness. At the interpersonal level, social support networks exert considerable influence. [20] examining psychological assistance services among older individuals in Turkish society found that marital status, living arrangements, and having confidants significantly predicted help-seeking. Their study revealed that individuals living alone or lacking trusted confidants were substantially less likely to seek psychological help, emphasizing that social isolation operates as an active barrier to accessing professional support. At the systemic level, healthcare factors create structural barriers including fragmented service delivery, insufficient integration between physical and mental healthcare, and limited specialist availability. [21] investigating mammography screening participation found that healthcare system factors; including provider encouragement and integration of preventive care into routine practice, significantly influenced health-seeking behaviors, illuminating parallel barriers in mental health service access.

The vast majority of mental health help-seeking literature originates from Western contexts where healthcare systems and cultural attitudes differ substantially from Asian settings. In collectivistic Asian societies, family-centered decision-making often predominates over individual autonomy in health-related choices. [22] found that Turkish individuals with mental health conditions face multiple barriers including stigma concerns, family attitudes, healthcare access challenges, and insufficient MHL, while also identifying important facilitators including family encouragement and healthcare provider recommendations. These findings from Turkish contexts, which share cultural similarities with Malaysia, including a collectivistic orientation and Islamic influences, suggest that culturally adapted interventions must address both universal help-seeking barriers and culture-specific factors.

The application of behavioral theories, particularly the Theory of Planned Behavior (TPB), has demonstrated reliable predictive validity for help-seeking intentions across diverse populations [23], though the intention-behavior gap remains a persistent challenge in translating help-seeking intentions into actual service utilization. The TPB posits that intentions are influenced by attitudes toward the behavior, subjective norms (SN) and perceived behavioral control (PBC) [24]. Complementing this the Health Belief Model (HBM) [25] emphasizes how individuals evaluate their susceptibility to health problems, the severity of consequences, and weigh perceived benefits against barriers when making health-related decisions. While TPB focuses on intentional aspects shaped by social and psychological factors, HBM addresses health-specific belief considerations including threat perception and cost-benefit evaluation. [22] applying multivariate probit regression found that health belief constructs—particularly perceived benefits and reduced perceived barriers—significantly predicted engagement with mental health professionals among Turkish individuals with mental health conditions. Integrating both frameworks provides comprehensive examination of help-seeking behavior.

Incorporating patient and healthcare provider perspectives offers a complete understanding. Patient perspectives illuminate internal processes and barriers; provider perspectives reveal systemic factors and intervention points. The Nominal Group Technique (NGT) provides rigorous consensus-building for prioritizing intervention-amenable factors. This complementary approach enables triangulation and multilevel intervention target identification.

This study makes several distinctive contributions to the mental health help-seeking literature. First, it represents the first investigation to systematically integrate the TPB and HBM through complementary qualitative approaches—combining in-depth patient interviews, Nominal Group Technique with healthcare professionals, and Fuzzy Delphi Method (FDM) validation. While previous studies have applied these frameworks quantitatively [11,12] our qualitative methodology provides deeper insights into lived experiences and decision-making processes. Second, it addresses a critical gap by triangulating patient and professional perspectives, whereas most existing research focuses exclusively on patients [26]. Third, it addresses geographical and cultural gaps, as the majority of help-seeking literature originates from Western contexts ([16,20,22]. By examining help-seeking within the Malaysian context—characterized by collectivistic orientation, Islamic influences, and multi-ethnic composition—our findings contribute culturally specific knowledge for Southeast Asian populations. Fourth, it focuses specifically on BC patients, a population facing unique psychological challenges yet underrepresented in mental health help-seeking research [2].

## Materials and methods

**Fig 1:**
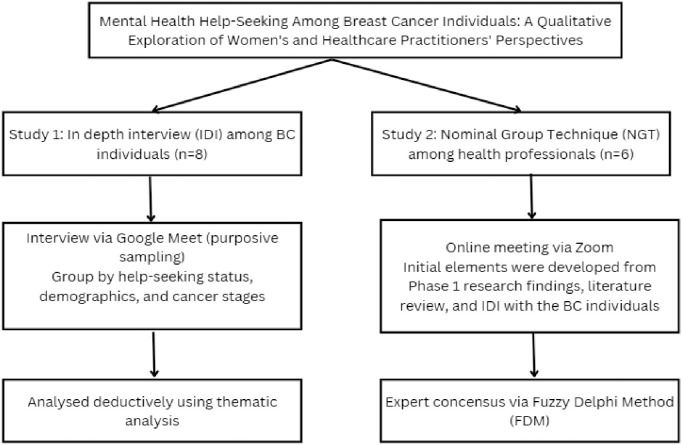
Study Design and Methodological Framework for Exploring Mental Health Help-Seeking Among BC Individuals.

This study employed purposive sampling to recruit 14 participants. Conducted between October 2024 and January 2025, the study integrated NGT with six healthcare professionals and IDIs with eight BC patients, guided by TPB and HBM frameworks. Fig 1 illustrates the sequential phases. The healthcare professionals were recruited from diverse healthcare settings across Malaysia, including government hospitals, teaching hospitals, private hospitals, the Ministry of Health, and government health clinics. Participants were identified through professional networks and direct invitation based on their expertise in BC management and patient care. The sample size of six experts was determined based on established NGT methodology guidelines, which recommend 5–9 participants as optimal for effective group interaction and consensus-building ([24,27]. Eligibility criteria included a minimum of 5 years of clinical experience in BC management and promotional activities, active involvement in direct patient care or patient support programs, and representation across multiple disciplines to capture diverse professional perspectives.

Eight individuals with BC were recruited from participants of a previous quantitative cross-sectional survey (n = 150) conducted by the research team between August and October 2024, which examined mental health help-seeking behaviors among BC patients using TPB and HBM frameworks [28]. The survey was administered at the National Cancer Institute (NCI), Malaysia, and through BC support groups on WhatsApp platforms. From the survey respondents who indicated willingness to participate in follow-up qualitative studies, purposive maximum variation sampling was employed to ensure diverse representation across three key dimensions. First, regarding help-seeking status, equal numbers of individuals who had sought professional mental health support (n = 4) and those who had not (n = 4) were recruited, enabling exploration of facilitators and barriers from both perspectives. Second, demographic characteristics were varied to include participants across a wide age range (37–65 years), different marital statuses (married and unmarried), and diverse education levels to capture different social contexts. Third, cancer stage representation spanned stages I-III to reflect different phases of the cancer journey and associated psychological challenges.

The sample size of eight participants was determined based on the concept of “information power” in qualitative research [29], which posits that sample size adequacy depends on five factors: [1] study aim—this study had a narrow, specific aim focused on help-seeking behavior; [2] sample specificity—highly specific (patients with diverse help-seeking experiences); [3] theoretical framework—analysis was guided by established theories [4] quality of dialogue—in-depth interviews facilitated rich, detailed accounts; and [5] analysis strategy—deductive thematic analysis provided clear analytical structure. Given the high information power achieved through these factors, a smaller sample size was appropriate and sufficient [30]. Data saturation, defined as the point at which no new themes or insights emerged from additional interviews, was monitored throughout the data collection process and was achieved after the eighth interview, confirming sample adequacy for the research objectives.

### Informed consent

All participants (both healthcare professionals and individuals with BC) provided written informed consent prior to participation. The consent process included detailed explanations of the study purpose, procedures, voluntary nature of participation, confidentiality measures, and the right to withdraw at any time without consequences. Confidentiality was maintained throughout the study by anonymizing all participant contributions in written materials, with participants identified only by coded identifiers in Table 1.

**Table 1:** Characteristics of participants in nominal groups and in-depth interview.

Group	Specification	n	Description
Healthcare Professionals^a^ (n = 6)	Psychiatrist (PSY)	2	PSY1: Government Hospital PSY2: Teaching Hospital
	Clinical Psychologist	1	Teaching Hospital
	Breast Surgeon	1	Private Hospital
	Public Health Specialist	1	Ministry of Health
	Family Medicine Specialist	1	Government Health Clinic
User^b^ (n = 8)	Seek Help	3	41 years old, Chinese, Married, Diploma, BC Stage II
			65 years old, Chinese, Married, High School Graduate, BC Stage II
			53 years old, Malay, Married, Degree, BC Stage I
	Do not seek help	5	37 years old, Malay, Married, Degree, BC Stage II
			49 years old, Chinese, Unmarried, Degree, BC Stage II
			51 years old, Sarawakian, Married, Degree, BC Stage III
			39 years old, Indian, Unmarried, Degree, BC Stage I
			57 years old, Malay, Married, High School Graduate, BC Stage III

Table 1 summarizes the participant groups, where it presents the distribution of healthcare professionals across different specialties and settings, and groups the individuals with BC by help-seeking status, demographics, and cancer stages.

### Study 1: IDIs

#### Tools and instrument.

The IDI protocol was adapted from [31], which uses a semi-structured interview approach commonly employed in qualitative research for exploring psychological help-seeking behaviors. The interview guide was structured around the integrated TPB and HBM theoretical frameworks, incorporating questions that explored six key constructs: (1) attitudes toward seeking mental health help, (2) SN regarding help-seeking, (3) PBC over accessing mental health services, (4) help-seeking intentions, (5) perceived barriers (self-stigma in seeking help), and (6) perceived benefits (MHL).

The IDI protocol was adapted from [31] and used a semi-structured interview approach commonly used in qualitative research. Potential participant passivity was addressed by incorporating stimulation questions (probes) into the protocol to facilitate conversation flow. Two qualitative research experts (a family medicine specialist and a psychiatrist) evaluated and endorsed the alignment of the protocol with the study objectives. Each interview lasted between 15 and 30 minutes, with all interviews completed within a 2-week period.

#### Data collection.

The IDIs were conducted virtually through Google Meet with eight BC individuals: those who sought and did not seek mental health support and individuals from various demographic backgrounds and cancer stages. Each participant was interviewed individually. The session began with the interviewer’s self-introduction and an explanation of the interview process. The participants provided consent for the recording and were required to enable their cameras during the interview, allowing the interviewer to observe behavioral expressions. All interview sessions were recorded, transcribed verbatim, and underwent thematic analysis.

#### Data analysis.

The data were analyzed using a deductive thematic approach. The qualitative content analysis proceeded according to the methodology described by Graneheim and Lundman [32], which involves systematically identifying codes, main themes, and sub-themes. Initially, the interview transcripts were reviewed thoroughly to gain familiarity and data immersion. The data were then coded based on emergent patterns using ATLAS.ti 7.msi software. The codes were subsequently examined to identify common elements, which were organized into main themes and sub-themes. The research team collectively reviewed and reached a consensus on all codes before finalizing the thematic structure. This rigorous process ensured the credibility of the results and alignment with the study objective.

### Study 2: NGT

#### Tools and instrument.

**Fig 2:**
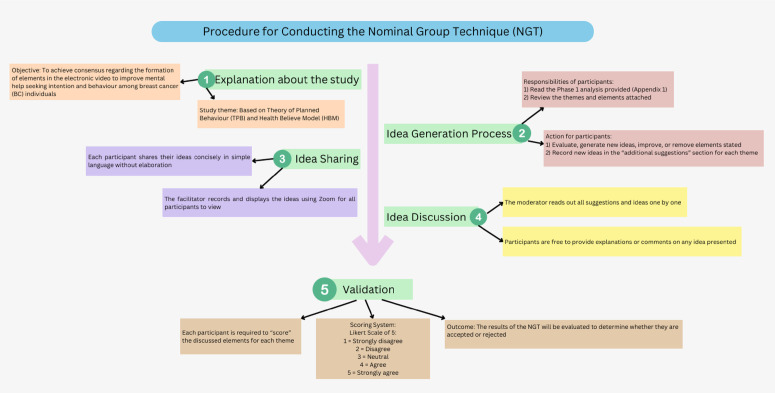
Schematic representation of the NGT protocol utilized in the current study.

The NGT sessions were structured using a standardized five-stage protocol adapted from [33] (Fig 2). The discussion framework was developed based on three primary sources: [1] findings from a previous quantitative survey of 150 individuals with BC examining TPB and HBM factors and help-seeking behaviors [2,28] comprehensive literature review of mental health help-seeking barriers and facilitators among cancer patients, and [3] preliminary findings from the IDI phase of this study.

The NGT framework systematically addressed each TPB and HBM construct, with an additional focus on “cues to action”—environmental or internal triggers that prompt help-seeking behavior. Healthcare professionals were presented with initial elements derived from the quantitative survey and asked to generate, discuss, and prioritize factors they considered most influential in promoting or hindering mental health help-seeking among individuals with BC.

#### Fuzzy Delphi Method (FDM) evaluation tool.

Following the NGT discussions, expert consensus on the identified elements was quantitatively assessed using the FDM. Participants rated each element on a 5-point Likert scale (1 = strongly disagree to 5 = strongly agree) regarding its importance in influencing mental health help-seeking behavior. The FDM analysis employed triangular fuzzy numbers to handle the inherent uncertainty and subjectivity in expert judgments [34].

An element was accepted as achieving expert consensus if it satisfied three established FDM criteria: [1] fuzzy evaluation score (A) > 0.5, indicating that the fuzzy mean exceeded the threshold value, [2] expert consensus percentage ≥ 67%, demonstrating substantial agreement among participants, and [3] threshold value (d) ≤ 0.2, indicating minimal dispersion between expert opinions [34]. This rigorous analytical approach ensured that only elements with strong expert validation were retained for interpretation.

### Data collection

Due to geographical constraints, the NGT sessions were conducted virtually via Zoom, with one facilitator, moderator, and observer to ensure productive engagement. The implementation adhered to a five-stage protocol (Fig 2), systematically addressing TPB and HBM constructs.

The NGT session began with the moderator briefly explaining the research objectives, followed by individual silent ideation, collaborative idea-sharing, concept refinement through addition, suggestion consolidation or elimination, and individual prioritization of key concepts through voting. The process concluded with a collective review of the aggregated rankings. All sessions were digitally recorded and subsequently transcribed for thorough analysis.

### Data analysis

The NGT generated two distinct data types: [1] written ideas and prioritization validated by the group independently, and [2] comprehensive discussions that clarified these ideas. The discussion data were merged and transcribed for analysis.

Based on the four TPB and two HBM constructs, the final items were analyzed using the FDM to establish expert consensus. The participants ranked the items generated during the NGT discussions using a 5-point Likert scale, and responses were processed using an FDM analysis template [35].

### Ethical consideration

This study was conducted with Medical Research Ethics Committee of Universiti Kebangsaan Malaysia approval (protocol code: JEP-2024–400; approval date: July 9 2024). All NGT and IDI participants provided informed written consent prior to participation. Confidentiality was maintained by anonymizing all participant contributions in written materials (Table 1).

## Results

### Study 1: IDIs

The IDIs obtained the participants’ views on the factors influencing their mental health help-seeking behavior based on the TPB and HBM constructs.

#### Attitude towards seeking help.

Most participants acknowledged the significant value of disclosing personal information to mental health professionals. Most participants expressed positive perspectives on sharing emotions and private details, emphasizing these professionals’ clinical expertise in assessment and intervention planning. (W7, 39, unmarried, stage I) articulated this sentiment clearly: “Sharing my emotions helps because the professional can assess the severity of my situation and identify appropriate solutions”.

Nevertheless, a minority of participants expressed reluctance or discomfort with emotional disclosure. One participant (W5, 49, unmarried, stage II) stated: “I prefer identifying the root cause of my problems and working through them independently”. These contrasting viewpoints highlighted the diverse help-seeking preferences among individuals with BC and underscored the importance of offering flexible, personalized approaches to psychological support.

#### Subjective norm.

The participants’ perceptions of societal views on psychological help demonstrated an evolution toward greater acceptance. Many participants noted that society has developed more positive attitudes toward seeking psychological help, with significant shifts in public understanding. One participant (W1, 41, married, stage II) observed this change directly: “Previously, people would label those seeking psychological help as ‘crazy’, but now seeking help is becoming more common”. Another participant (W6, 51, married, stage III) supported this perspective: “Society has developed a more positive outlook nowadays, with a better understanding of individual support needs”.

However, concerns regarding stigma persist, with some participants expressing apprehension about negative social judgments. One respondent (W3, 53, married, stage I) shared: “I worry about the societal stigma that labels people seeking psychological help as mentally ill. Therefore, I don’t share with others that I’m getting psychological support”. Another participant (W7, 39, unmarried, stage I) expressed similar concerns: “In my experience, people often misinterpret those seeking psychological help as either ‘crazy’ or attention-seeking. That’s how they perceive it when someone is depressed”.

#### Perceived behavioral control.

Various barriers to help-seeking were identified. These included emotional threshold recognition (seeking help when emotions become unmanageable), facilitating factors such as supportive friends, professional assurance of confidentiality, and circumstantial triggers. One participant (W8, 57, married, stage III) clearly articulated her personal threshold: “I would think about getting psychological help if my emotions become unstable, though right now I can still manage them”.

Personal experiences significantly influenced help-seeking decisions. One participant (W2, 65, married, stage II) shared a specific circumstance that prompted her to seek help: “I was relying on sleeping pills to get any rest, and my friend encouraged me to talk to a psychiatrist about it”. Another participant (W7, unmarried, stage I) highlighted the role of support systems: “My family has been very supportive throughout my cancer journey, encouraging me to stay active and join support groups”.

#### Help-seeking intention.

The participants demonstrated mixed intentions toward seeking psychological help. Some respondents expressed self-sufficiency, preferring to cope independently with their situations: “For now, I haven’t felt the need for psychological support as I can still cope with my situation” (W4, 37, married, stage II). Others denied needing help despite experiencing emotional distress related to their health journey: “I don’t feel I need psychological help because my emotional state isn’t severely affected by my BC journey” (W8, 57, married, stage III).

Several participants demonstrated emotional avoidance, particularly avoiding discussions about emotions with professionals: “I prefer not to discuss my emotions with experts. I try to stay positive and find my own ways to cope with the challenges” (W5, 49, unmarried, stage II). However, some participants acknowledged having needed psychological support in the past, noting the importance of family and friends as informal support systems: “I haven’t needed psychological support because my family and friends provide me with strong emotional support” (W6, 51, married, stage III).

#### Perceived barriers (self-stigma in seeking help).

Various barriers to help-seeking were identified. These included perceived lack of need, with participants reporting sufficient informal support: “Currently, I don’t feel the need for psychological support as I receive good support from my family and friends” (W4, 37, married, stage II).

Stigma-related concerns about others’ perceptions were prominent: “The main factors are embarrassment and concerns about people’s perceptions, which could affect my career” (W3, 53, married, stage I). The participants also mentioned access uncertainty: “I was uncertain about where to find help and its effectiveness” (W1, 41, married, stage II). Additionally, social support issues created barriers: “My spouse’s disbelief about my anxiety creates a barrier to seeking help” (W2, 65, married, stage II). Collectively, these factors impeded the participants’ seeking professional psychological support even when potentially beneficial.

#### Perceived benefits (MHL).

The participants were asked, “In your opinion, in which situation does an individual need to seek psychological help?” and identified several circumstances warranting professional psychological intervention. The participants recognized that the key indicators thereof were emotional deterioration, lack of support, behavioral warning signs, and crisis points. Specific indicators included emotional changes: “When there’s a noticeable change in emotional state, like shifting from being cheerful to becoming withdrawn and directionless” (W3, 53, married, stage I). Other warning signs included sleep difficulties and behavioral changes: “When they experience sleep difficulties, overthinking, social withdrawal, and exhibit unusual behavioral changes” (W2, 65, married, stage II), and suicidal thoughts: “When someone lacks support systems and someone to talk to, this could lead to depression and suicidal thoughts” (W7, 39, unmarried, stage I).

Regarding professional roles, the participants demonstrated varied knowledge regarding the differences between psychiatrists, psychologists, and counsellors, with the following illustrating the most common understanding: “Psychiatrists prescribe medicines. Psychologists talk through issues, assess you, and determine treatment. Counsellors discuss problems and suggest solutions like breathing exercises” (W2, 65, married, stage II). Table 2 summarizes the systematic coding process and formation of sub-themes based on the integrated TPB and HBM theoretical constructs.

**Table 2:** Theme, subtheme and code identified from IDI among BC individuals (n = 8).

Theme (TPB and HBM Domain) *IDI protocol	Subtheme	Code
Help-Seeking Intention * Have you ever felt the need to receive psychological help before? Why?	Self – Sufficient	Believe in self-coping Reluctant to seek help
	Symptom recognition	Distress minimization Denying the need for help
	Preference on non-professional	Informal support system Emotional avoidance
Perceived Behavior Control * What makes it easier for you to seek psychological help? Anything that can influence your decision to seek help?	Factors that help in seeking support	Emotional threshold recognition Facilitating factors Professional assurance Circumstantial triggers
Attitude * When trying to overcome an emotional problem, is sharing private information about your problem with a mental health professional helpful?	Thoughts on opening up	Perceived benefits of disclosure Professional assessment value Disclosure hesitation Help-seeking rationalization
Subjective Norm *In your opinion, what does society think about people receiving psychological help for interpersonal or emotional problems?	Evolving societal perspectives on psychological help	Changing social views Persistent stigma Increased public understanding Mixed social acceptance
Perceived Barriers * Could you mention the factors that prevent/have prevented you from receiving psychological help?	Multifaceted barriers to help-seeking	Perceived lack of need Social support sufficiency Access uncertainty Stigma-related concerns
Perceived Benefits * In your opinion, in which situation does an individual need to seek psychological help?	When to seek help	Emotional deterioration Lack of support Behavioral warning signs Crisis points
Perceived Benefits *What do a psychiatrist, a clinical psychologist, and a counsellor do? Do you know the differences between these professions?	Knowledge about mental health professionals	Role recognition Knowledge gaps Service differentiation

### Study 2: NGT

The FDM evaluation demonstrated strong expert acceptance across all assessed elements influencing mental health help-seeking behaviors among individuals with BC. The analysis identified particularly high expert agreement in areas related to emotional support mechanisms, with the elements focusing on building emotional strength through help-seeking and family support systems receiving the highest fuzzy scores. Expert consensus was 100% for most elements, demonstrating clear agreement regarding the importance of these factors in promoting mental health help-seeking behaviors.

The evaluation highlighted the significance of individual and systemic factors in facilitating help-seeking behaviors. At the individual level, the critical components were understanding mental health resources and having autonomy in decision-making. At the systemic level, the key enablers of help-seeking behavior were social support networks, including family members, cultural leaders, and healthcare professionals. The importance of fostering trust in mental health providers and normalizing mental health support within regular clinical practice also received strong expert endorsement, indicating the value of integrated approaches to mental healthcare for individuals with BC. Table 3 presents the final consensus among the health professionals (n = 6) based on the FDM assessment. The table identifies and ranks the most significant elements within the TPB and HBM constructs that influence mental health help-seeking behaviors in individuals with BC.

**Table 3:** Health professionals (n = 6) consensus using FDM assessment.

Video content elements based on TPB and HBM	Triangular Fuzzy Number		Fuzzy Evaluation Process				Overall Expert Acceptance	Ranking
	Threshold Value (d)	Expert Consensus (%)	m1	m2	m3	Fuzzy Score (A)		
**Attitude towards seeking help**								
Understanding help-seeking builds emotional strength, improves attitudes toward support	0.080	100.0%	0.708	0.958	1.000	0.889	Accepted	1
Cancer survivors’ testimonials foster help-seeking attitudes	0.205	83.3%	0.625	0.875	0.958	0.819	Accepted	2
Influence from celebrities/famous people regarding the benefits of seeking mental health support improves help-seeking attitude	0.239	33.3%	0.500	0.750	0.917	0.722	Rejected	3
Suggestion to seek mental health support as a form of rejection by the primary team improves help-seeking attitudes (“I was asked to see a psychiatrist because they were annoyed with my anxiety” (Ms H, 62, stage I))	0.460	16.7%	0.292	0.500	0.667	0.486	Rejected	4
**Subjective Norm**								
Support from family members increases willingness to seek psychological help (“My husband doesn’t believe that I have this mental problem. So, I cannot seek help” (Ms. G, 41, stage II))	0.128	100.0%	0.708	0.917	1.000	0.861	Accepted	1
Open mental health discussions on social media enhance support acceptance	0.128	100.0%	0.583	0.833	1.000	0.806	Accepted	3
Cultural and religious leaders’ advocacy strengthens community help-seeking acceptance	0.144	100.0%	0.625	0.875	1.000	0.833	Accepted	2
Healthcare providers’ recommendations strengthen help-seeking decisions	0.286	66.7%	0.417	0.667	0.875	0.653	Rejected	4
**Perceived Behavioral Control**								
Understanding how to access mental health support step-by-step builds confidence in seeking help	0.128	100.0%	0.667	0.917	1.000	0.861	Accepted	1
Having the autonomy to decide through shared decision-making rather than patriarchal practice of advising	0.128	100.0%	0.667	0.917	1.000	0.861	Accepted	1
Having flexible online and telehealth options increases confidence in accessing care	0.144	100.0%	0.625	0.875	1.000	0.833	Accepted	3
Understanding professional expertise builds confidence in seeking help (“Yes it will be good to share because if you do share your problem with a professional, at least that person will know at what stage is your problem” (Puan S, 39, stage I))	0.269	66.7%	0.542	0.792	0.917	0.750	Rejected	4
The need for patient initiation in requesting mental health assessments affects sense of control over accessing support (“When we go to health clinics, this isn’t a normal procedure meaning they don’t give these assessments to everyone. Only if we say we want to do it” (Pn N, 37, stage II))	0.297	66.7%	0.375	0.625	0.833	0.611	Rejected	5
**Intention to seek help**								
Recognizing personal stress limits increases willingness to seek professional support (“If I find myself unable to handle my stress during this journey, then I would seek help” (Ms. C, 49, stage II))	0.481	83.3%	0.625	0.875	0.958	0.819	Accepted	2
The level of intent to do whatever it takes to feel better increases willingness to seek professional mental health support	0.144	100.0%	0.625	0.875	1.000	0.833	Accepted	1
Mental health prioritization reinforces help-seeking intentions	0.419	66.7%	0.583	0.833	0.917	0.778	Rejected	3
**Perceived Barriers (Self-Stigma Towards Seeking Help)**								
Viewing mental health care as important to physical care reduces internal stigma	0.144	100.0%	0.625	0.875	1.000	0.833	Accepted	2
Developing trust with mental health providers helps overcome barriers to disclosure (“I don’t think it’s hard, but some people will be reluctant in the beginning but you need to win their trust” (Ms. S, 39, stage I))	0.128	100.0%	0.583	0.833	1.000	0.806	Accepted	1
Professional role concerns and fear of workplace stigma create barriers to seeking support (“When they said I need to see a counsellor, I worried about it affecting my service record. As I work in healthcare, I don’t want people to think about me that way” (Ms. J, 53, stage I))	0.144	100.0%	0.625	0.875	1.000	0.833	Accepted	2
**Perceived Benefits (MHL)**								
Understanding mental health increases comfort in getting professional help	0.192	100.0%	0.667	0.917	1.000	0.861	Accepted	1
Lack of information about available resources creates barriers to accessing mental health support (“I don’t know where to seek help at that time” (Ms. G, 41, stage II))	0.192	100.0%	0.667	0.917	1.000	0.861	Accepted	1
Health professionals prioritizing/normalizing mental health support in clinical practice	0.192	100.0%	0.667	0.917	1.000	0.861	Accepted	1
**Cue to Action**								
Family-targeted health information activates help-seeking through family support	0.144	100.0%	0.625	0.875	1.000	0.833	Accepted	1

## Discussion

This study makes several original contributions to understanding mental health help-seeking among breast cancer patients. To our knowledge, this is the first qualitative investigation to systematically integrate TPB and HBM frameworks through complementary methodologies, specifically combining in-depth patient interviews with NGT involving healthcare professionals and FDM validation. While previous studies have predominantly used quantitative approaches to identify correlates [11,12], our qualitative approach provides deeper insights into lived experiences and decision-making processes underlying these statistical relationships.

The triangulation of patient and professional perspectives represents a significant methodological advancement. Most existing research focuses exclusively on patient perspectives [36], potentially overlooking systemic factors and intervention opportunities visible to healthcare providers. Our findings reveal both convergent perspectives (family support importance, stigma impact) and divergent views (autonomy in decision-making), highlighting the value of incorporating multiple stakeholder viewpoints. Furthermore, the application of FDM to quantify expert consensus on qualitative findings provides a rigorous approach to prioritizing intervention targets. This methodological integration is relatively novel in help-seeking research and demonstrates pathways for translating qualitative insights into actionable strategies.

Importantly, this study addresses critical gaps in geographical and cultural representation. The vast majority of existing literature originates from Western contexts, where healthcare systems, cultural attitudes, and social support structures differ substantially from Asian settings [16,20,22]. By examining help-seeking within the Malaysian context, our findings contribute culturally specific knowledge essential for developing contextually appropriate interventions in Southeast Asian populations.

### Attitude towards seeking help

The paradox between recognizing professional support value and personal reluctance aligns with [37], who observed that theoretical knowledge fails to translate into behavior due to psychological barriers. This finding extends recent work by [16], who documented that positive attitudes toward help-seeking do not automatically translate into service utilization, particularly among individuals experiencing high levels of self-stigma or social pressure. Our findings reveal that attitudes toward help-seeking are shaped by multiple interacting factors including prior experiences with healthcare systems, quality of existing informal support networks, and culturally-influenced beliefs about appropriate coping strategies. This aligns with [22] documentation that help-seeking attitudes among Turkish individuals with mental health conditions were significantly influenced by social support availability, healthcare system experiences, and cultural norms regarding mental illness. The similarity suggests that these factors may operate across diverse Asian, Islamic, and collectivistic cultural settings. The NGT results showing that understanding help-seeking builds emotional strength suggest interventions must address not only awareness but also internalized stigma [38]. Cancer survivor testimonials also emerged as powerful influences, consistent with peer narrative interventions reducing stigma [39]. This suggests combined interventions addressing emotional benefits and peer testimonials may effectively transform attitudes toward psychological support [40].

### Subjective norm

Social and cultural norms significantly influenced help-seeking, consistent with [41]. Despite these positive trends, the IDI results revealed enduring stigma concerns, with participants expressing apprehension regarding negative social judgments that could result from seeking psychological support. This result is consistent with previous research demonstrating how perceived social approval significantly influences help-seeking intentions among individuals with cancer [8]. This tension between improved social acceptance and persistent stigma creates a complex normative environment that significantly influences help-seeking decisions.

NGT results showed family support as the most critical factor increasing help-seeking willingness, receiving 100% expert consensus. This finding aligns with [20] identification of family support as a primary determinant of psychological help-seeking among older adults. Their study documented that individuals lacking close family relationships or confidants demonstrated substantially lower help-seeking rates even when experiencing significant psychological distress. Our findings extend this understanding to BC populations, revealing that family influence operates through multiple pathways: providing informal emotional support (which may substitute for professional services), actively encouraging or discouraging formal help-seeking, and shaping perceptions of social acceptability of mental health service use.

Cultural and religious leaders’ advocacy also strengthens community acceptance, reflecting significant influence in value-driven communities [42]. This finding parallels [22] observation that community-level factors, including endorsement by respected authority figures, significantly predict mental health service utilization. The convergence of these findings underscores the importance of engaging cultural and religious leaders in intervention efforts within Islamic and collectivistic societies. Additionally, open mental health discussions on social media were recognized as enhancing support acceptance, which was consistent with [43], who demonstrated how digital platforms can reshape mental health norms among individuals with cancer. These results suggest interventions targeting the social environment at multiple levels, from family to community leaders to broader social media discourse, may be more effective than those focusing solely on individual factors.

### Perceived behavioral control

PBC findings revealed critical factors influencing help-seeking ability: emotional threshold recognition, facilitating factors, professional assurance, and circumstantial triggers [36]. This pattern creates significant barriers to timely support, potentially allowing distress to escalate before intervention, consistent with findings that delayed help-seeking worsens outcomes.

NGT findings highlighted practical elements enhancing perceived control. “Understanding step-by-step access to mental health support” received unanimous endorsement (100% consensus, first rank), directly addressing process uncertainty—a well-documented barrier [44]. This finding resonates with [21] observation that clear information about healthcare service access processes significantly influences health-seeking behaviors, including mammography screening participation. While their study focused on cancer screening rather than mental health services, the parallel suggests that procedural clarity represents a generalizable facilitator across diverse health-seeking contexts. “Having autonomy through shared decision-making” received equal importance (100% consensus), underscoring patient agency and aligning with evidence that shared decision-making improves engagement [45]. “Flexible online and telehealth options” also increased confidence, reflecting evolving healthcare preferences where telehealth has gained wider acceptance [46].

These findings suggest interventions should address access knowledge gaps, decision-making autonomy, and flexible delivery options accommodating patient preferences.

### Help-seeking intention

Help-seeking intentions varied considerably, with prominent self-reliance themes representing significant barriers to professional help-seeking, consistent with broader literature on cancer patients’ coping strategies [47]. Our findings reveal that self-reliance is not merely an individual personality trait but is actively reinforced by cultural values emphasizing emotional restraint, family solidarity, and aversion to external help-seeking that might reflect poorly on one’s family.

NGT findings identified “intent to do whatever it takes to feel better” as increasing willingness to seek support, suggesting well-being-focused intrinsic motivation may be more effective than problem-oriented framing [48]. This finding extends [16] observation that help-seeking intentions are stronger when framed around personal growth and wellness rather than illness and deficit. Intervention messaging emphasizing empowerment, wellness, and quality of life enhancement may be more effective than messages emphasizing mental health problems requiring treatment.

NGT findings also identified “recognizing personal stress limits,” aligning with the emotional threshold concept. This conditional intention indicates openness to seeking help under certain circumstances, though potentially delaying intervention until significant distress levels [49].

### Perceived barriers (self-stigma in seeking help)

Perceived barriers analysis revealed significant internal challenges. Informal support networks profoundly influenced decisions, with many citing family and friends as primary psychological resources. While strong social support correlates with better outcomes [50], exclusive reliance on informal networks may prevent access to evidence-based interventions during critical periods. This finding aligns with [20] observation that individuals with strong informal support networks may paradoxically demonstrate lower formal help-seeking rates, potentially due to perceived adequacy of informal support or concerns about disrupting valued relationships by seeking external help. Participants acknowledging emotional difficulties yet remaining reluctant suggest psychological barriers like self-stigma and emotional avoidance exceed practical access challenges [51]. This recognition-reluctance paradox represents a critical finding extending existing help-seeking literature. [22] work documented similar patterns among individuals with mental health conditions, finding that self-stigma and anticipated discrimination predicted help-seeking reluctance even when individuals possessed adequate mental health literacy and positive general attitudes toward mental health services.

Career-related concerns and workplace stigma emerged as significant barriers. Professional role concerns and fear of workplace stigma create substantial barriers, particularly for healthcare workers where professional identity intensifies stigma concerns [52].

NGT findings showed “developing trust with mental health providers” as important for addressing self-stigma. Patients developing provider confidence become more willing to disclose concerns and overcome barriers [53], aligning with evidence that early therapeutic alliance reduces reluctance and improves engagement.

Another key NGT result was that “viewing mental health care as important to physical care reduces internal stigma”. This integration of mental and physical healthcare represents a potential pathway to normalize psychological support in cancer treatment [9]. This result suggested that integrated care models that present psychological support as a standard component of comprehensive cancer treatment may aid in reducing self-stigma and increase help-seeking behaviors.

### Perceived benefits (MHL)

MHL analysis revealed varying understanding of professional intervention circumstances. Participants identified emotional deterioration, behavioral warning signs, and crisis points requiring help. Role understanding inconsistency indicated poor literacy potentially causing confusion and unrealistic expectations [54].

NGT identified that understanding mental health increases comfort (consistent with improved literacy reducing discomfort), while lack of resource information creates barriers. Health professionals prioritizing mental health support in clinical practice emerged as crucial for validating concerns and facilitating behaviors in standard cancer care [10]. This finding aligns with broader evidence that provider recommendations represent one of the strongest predictors of health service utilization across diverse healthcare contexts [21]. A significant NGT result was the importance of “health professionals prioritizing/normalizing mental health support in clinical practice”. This result indicated that healthcare providers are crucial in validating mental health concerns and facilitating help-seeking behaviors in standard cancer care [55].

### Cue to action

The NGT identified “family-targeted health information activates help-seeking through family support” as an important triggering mechanism for cue to action. This result suggested that educational interventions directed at patients and their family members may be particularly effective in prompting help-seeking behaviors. Equipping families with appropriate knowledge and resources can be key motivators for encouraging professional support when needed [55]. This finding extends the HBM’s cue to action construct by documenting that cues operating through trusted social networks may be more powerful than cues originating from healthcare providers or mass media campaigns. In collectivistic contexts where family relationships are central, leveraging family members as agents of change may represent a culturally appropriate and particularly effective intervention strategy.

These complementary methodologies (IDI and NGT) provide a holistic understanding of mental health help-seeking among individuals with BC that neither approach could achieve alone. While the IDIs revealed complex psychological barriers and personal decision-making processes, the NGT results identified evidence-based elements that health professionals consider most effective in promoting help-seeking behaviors. This integrated perspective suggested that effective interventions must operate at multiple levels simultaneously: addressing individual psychological barriers through targeted education and stigma reduction, strengthening the capacity of family systems to encourage appropriate professional help-seeking, and transforming healthcare delivery to normalize psychological support as an integral part of cancer care. By addressing both the lived experiences uncovered through IDIs and the expert-validated factors identified through NGT, interventions can more effectively bridge the gap between recognizing the value of mental health support and engaging with these essential services during the cancer journey.

### Contribution and implications

This study provides empirical support for the complementary application of TPB and HBM in understanding help-seeking behavior, demonstrating how these theories capture different but interconnected aspects of decision-making. Our methodological contribution of combining patient narratives (IDI) with professional consensus-building (NGT) and expert validation (FDM) offers a replicable model for bridging understanding of patient experiences and developing professionally validated intervention strategies.

A key theoretical contribution is our documentation of the “recognition-reluctance paradox”; wherein participants acknowledged the value of professional psychological support while remaining reluctant to personally engage with these services. This paradox extends existing understanding of the help-seeking treatment gap by revealing that the barrier is not merely lack of awareness, but rather powerful influences of self-stigma, emotional avoidance, and preference for informal support networks. This has important intervention design implications, suggesting educational approaches alone are insufficient and must couple with strategies addressing internalized stigma and normalizing help-seeking within cancer care.

The study contributes to cultural factors literature by documenting how collectivistic values influence help-seeking in Asian contexts, where family support operates at multiple levels; providing informal emotional support that may substitute for professional help while also actively influencing formal service decisions. This underscores the need for family-inclusive intervention approaches in Asian populations. Comparisons with recent work [16,20,22] revealed similarities including strong family influence, persistent stigma, and healthcare access challenges, strengthening the cross-cultural validity of these findings.

Our findings provide practical healthcare system improvement knowledge by identifying specific, evidence-based intervention points. Strong expert consensus on integrating mental health support into routine oncology care, providing clear access pathways, and enabling shared decision-making offers actionable guidance for administrators and policymakers seeking improved psychological support utilization. Specifically, our results suggest several practical intervention strategies: [1] routine psychological screening integrated into standard oncology visits, [2] family-inclusive psychoeducation, [3] clear navigation support, [4] shared decision-making approaches, and [5] flexible service delivery modalities including telehealth options.

### Study limitation

This study has limitations requiring acknowledgment. The sample was drawn exclusively from Malaysian BC patients accessing NCI care and support groups, potentially limiting transferability to other contexts. Purposive sampling and eight-participant IDI size, while justified by information power and saturation principles, precludes statistical generalization. All IDI participants previously participated in quantitative surveys, potentially introducing selection bias toward more health-literate individuals.

Cross-sectional data collection captured single-point perspectives, whereas attitudes and behaviors may evolve across cancer trajectories. Longitudinal research would provide richer insights into perspective changes. Virtual platforms (Google Meet, Zoom), while necessary for accessibility, may have limited interpersonal connection depth compared to in-person interactions. The six-participant professional sample, though multidisciplinary, focused on perspectives regarding patient help-seeking rather than direct provider-patient interaction observation.

Despite these limitations, this study provides valuable insights into the multifaceted factors influencing mental health help-seeking among individuals with BC through the complementary use of patient and professional perspectives, contributing meaningful knowledge for developing contextually appropriate interventions to improve psychological support utilization.

## Conclusion

Results revealed a complex interplay between individual psychological barriers and systemic factors that affect help-seeking decisions. The individuals with BC demonstrated awareness of the value of professional psychological support while frequently remaining reluctant to engage with these services due to emotional avoidance, self-stigma, and reliance on informal support networks. The health professionals reached a strong consensus on the elements that could effectively promote help-seeking, particularly those focused on normalizing mental health support, enhancing family involvement, and providing clear pathways to access care. These results suggested that effective interventions must address both individual psychological barriers and systemic healthcare delivery factors while respecting the autonomy of individuals with BC regarding their coping choices. Future research should focus on developing and evaluating multi-component interventions that incorporate the evidence-based elements identified through this study to bridge the gap between acknowledging the importance of mental health support and accessing these essential services during the cancer journey.
